# Toxin ζ Reduces the ATP and Modulates the Uridine Diphosphate-N-acetylglucosamine Pool

**DOI:** 10.3390/toxins11010029

**Published:** 2019-01-09

**Authors:** María Moreno-del Álamo, Mariangela Tabone, Juan Muñoz-Martínez, José R. Valverde, Juan C. Alonso

**Affiliations:** 1 Department of Microbial Biotechnology, Centro Nacional de Biotecnología, CNB-CSIC, 3 Darwin Str., 28049 Madrid, Spain; mmoreno@cnb.csic.es (M.M.-d.Á.); mariangelatabone@gmail.com (M.T.); 2 Scientific Computing Service, Centro Nacional de Biotecnología, CNB-CSIC, 3 Darwin Str., 28049 Madrid, Spain; juanmm.ah@hotmail.com

**Keywords:** Toxin-antitoxin system, cell wall inhibition, bacterial persistence, nucleotide hydrolysis, uridine diphosphate-N-acetylglucosamine

## Abstract

Toxin ζ expression triggers a reversible state of dormancy, diminishes the pool of purine nucleotides, promotes (p)ppGpp synthesis, phosphorylates a fraction of the peptidoglycan precursor uridine diphosphate-N-acetylglucosamine (UNAG), leading to unreactive UNAG-P, induces persistence in a reduced subpopulation, and sensitizes cells to different antibiotics. Here, we combined computational analyses with biochemical experiments to examine the mechanism of toxin ζ action. Free ζ toxin showed low affinity for UNAG. Toxin ζ bound to UNAG hydrolyzed ATP·Mg^2+^, with the accumulation of ADP, P_i_, and produced low levels of phosphorylated UNAG (UNAG-P). Toxin ζ, which has a large ATP binding pocket, may temporally favor ATP binding in a position that is distant from UNAG, hindering UNAG phosphorylation upon ATP hydrolysis. The residues D67, E116, R158 and R171, involved in the interaction with metal, ATP, and UNAG, were essential for the toxic and ATPase activities of toxin ζ; whereas the E100 and T128 residues were partially dispensable. The results indicate that ζ bound to UNAG reduces the ATP concentration, which indirectly induces a reversible dormant state, and modulates the pool of UNAG.

## 1. Introduction

Bacteria sense and respond to environmental stress with responses that can require dramatic cellular reprogramming. Protein toxins, of the toxin-antitoxin (TA) modules, are implicated in multiple cellular functions and are associated with cell survival under different stress conditions. Toxins and their cognate antitoxins have been detected in the chromosomes of bacteria, their phages and low-copy number plasmids, as well as in archaea, with more than 6000 putative modules identified so far [1]. The nature and activity of the antitoxins was used to classify the TA systems into six different types (I to VI). The largest group is formed by type II TAs [2]. In the type II TAs, which comprise a pair of gene-coding proteins, the antitoxin forms a tight complex avoiding the action of the toxin [2,3,4,5]. In the presence of stress, the unstable antitoxin is rapidly degraded, freeing the more stable toxin. Then, the free active type II toxin targets essential cellular processes such as RNA, DNA and protein synthesis, cell division, etc., and reversibly induces inhibition of cell proliferation (dormant state) [2,3,4,5,6,7,8,9]. These TAs may also contribute to antibiotics persistence [2,6,10,11], although a direct link between induction of TA systems and persistence to antibiotics has recently been challenged [12]. Type II toxins of the ζ superfamily, which are among the most broadly distributed in nature, sensitize bacterial cells to different antibiotics [13,14,15,16]. To understand how the toxin ζ might contribute to reducing recalcitrant and recurring infections, we have examined its molecular mechanism of action.

The TA cassette of the ζ-ε superfamily, which has been found in major human and plant pathogens, is phylogenetically well conserved [17,18]. In Firmicutes, toxin ζ from *Streptococcus pyogenes*, *S. agalactiae*, *Enterococcus faecalis*, *Clostridium perfringens* or *Staphylococcus aureus* (~285 amino acids) share ~43% sequence identity with the shorter *S. pneumoniae* PezT or *S. suis* SezT toxin (~255 amino acids) [18,19,20]. In solution, two ζ or PezT monomers interact with their cognate antitoxin dimers (ε_2_/PezA_2_), with ATP·Mg^2+^ (denoted as ATP) and with its target uridine diphosphate-N-acetylglucosamine (UNAG), which is an essential precursor of bacterial cell wall biosynthesis [19,21,22,23]. The interaction of toxin and antitoxin leads to the formation of a biological non-toxic hetero-tetrameric (ζε_2_ζ/PezT-PezA_2_-PezT) complex. The structure of the inactive ζε_2_ζ complex, both alone or UNAG bound, has been reported [19,21,22,23]. In these structures, the ATP binding site has been putatively assigned based on similarity to chloramphenicol phosphotransferase [19,22]. The crystal structure of the ζε_2_ζ TA complex and the complex bound to UNAG showed that the ATP binding pocket of the ζ/PezT toxin is sterically blocked by the binding of the ε_2_/PezA_2_ antitoxin, but the antitoxin cannot interfere with UNAG binding [19,21,22,23]. Therefore, the interactions with ATP and ε_2_/PezA_2_ are mutually exclusive [19,22].

In the presence of UNAG, toxin ζ primarily hydrolyses ATP rather than another nucleotide cofactors [24,25]. Toxin ζ, which fails to undergo auto-phosphorylation [24], transfers the ATP γ-phosphate (Pγ) to a fraction of the C3′-OH group of the amino sugar of the N-acetylglucosamine moiety of the peptidoglycan precursor UNAG amino sugar, producing irreversibly unreactive UNAG-P [23,24]. UNAG-P cannot be utilized by the MurA (MurAA and MurAB) enzyme(s) that catalyze the transfer of enolpyruvate from phosphoenolpyruvate to the C3′-OH group of the N-acetylglucosamine moiety of UNAG [23]. Thus, the blockage of the first committed step in the biosynthesis of peptidoglycan should lead to loss of cell shape and integrity [18,26]. This led to the proposal that toxin ζ might act by irreversibly depleting the pool of UNAG followed by bacterial cell lysis and death [23]. However, toxin ζ action, at or near physiological concentration, showed that it is reversible by nature, even in distantly related bacteria, such as *Escherichia coli* and *Bacillus subtilis* [13,27]. At or near physiological concentrations, toxin ζ reversibly halts *B. subtilis* proliferation rather than triggering cell lysis [13,14,25]. Furthermore, upon ζ expression, a large fraction of cells are still sensitive to fosfomycin, an antibiotic which selectively inhibits MurA activity [26], suggesting that only a fraction of the UNAG pool is irreversibly phosphorylated in vivo by toxin ζ [24]. A deeper understanding of the mechanism of ζ action, which inhibits cell proliferation by interfering with vital processes and sensitizes bacterial cells to different antibiotics [13,14,15], will greatly facilitate targeted engineering of its activity as a potent antimicrobial.

In this study, the interactions of toxin ζ or its variants with ATP and UNAG have been subjected to detailed computational and biochemical analyses to investigate how ζ controls UNAG phosphorylation and how this process may contribute to stress control. A quantitative analysis of the reaction reveals that ζ can hydrolyze > 95% of ATP in the reaction, but under these conditions, only a small fraction of UNAG (up to 7%) is phosphorylated. Computer modeling was built up to explain why the transfer of the phosphate is so inefficient. These findings led us to propose that toxin ζ, by hydrolyzing ATP, induces a metabolic ATP/GTP imbalance. This leads to altered gene expression with increased (p)ppGpp synthesis, which reduces the pool of purine nucleotides and halts cell proliferation to cope with the stress [13,14,25]. Complementarily, toxin ζ phosphorylates a fraction of UNAG, leading to unreactive UNAG-P, rather than depleting the UNAG pool.

## 2. Results

### 2.1. Toxin ζ Has a Large ATP Site Pocket

Crystallographic data are available for the apo *ζε*_2_*ζ* complex (1GVN) and for this complex bound to UNAG (3Q8X) [19,22,23]. In both structures, the C-terminal residues of the *ζ* polypeptide chains cannot be seen in the electron density maps, implying a high degree of flexibility. To understand the properties of the *S. pyogenes* toxin *ζ*, especially its unassigned C-terminal region, and its ATP bound form, we performed a computational analysis. The model for full-length toxin *ζ* was built using iterative threading as implemented in the I-TASSER suite [28]. According to this model, the missing C-terminal region might fold in the face opposite to the antitoxin *ε*_2_ binding site, and the putative ATP binding site, and therefore it would be unlikely to interfere with toxin *ζ* activity. This is consistent with the observation that a PezT variant lacking the unassigned C-terminal end is an active toxin [23].

**Figure 1:**
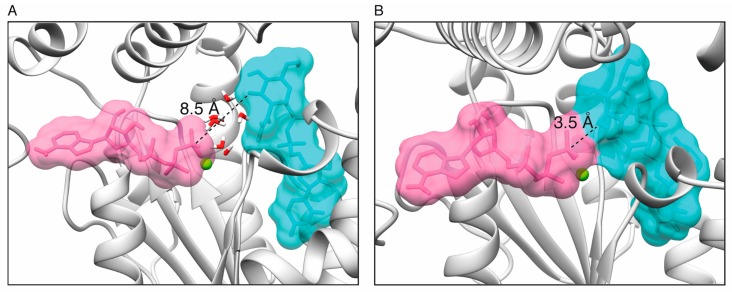
The UNAG and ATP pockets. The two main pockets identified by 3v superimposed to ζ, UNAG and the predicted binding site for ATP·Mg^2+^. Toxin ζ active sites with ATP (purple) located in extreme positions in the binding pocket, either far (~8.5 Å, (**A**)) or close (~3.5 Å, (**B**)) to the O3’ of UNAG (cyan). The Mg^2+^ ion is denoted as a green ball.

The ATP binding site in the *ζ* toxin was initially assigned based on similarity to genuine phosphotransferases [19,22]. Pocket predictions using 3v [29] showed only two putative pockets: the one corresponding to the known UNAG binding site, and a larger one, where the N-terminal end of the *ε*_2_ antitoxin binds in the crystal structure [19,22]. This is the proposed binding location for ATP (Figure 1A,B). The I-TASSER modeled toxin *ζ* structures were superposed with the known structures of related phosphotransferases as chloramphenicol phosphotransferase (1QHX) [30], O-phosphoseryl-tRNA kinase (3AM1) [31] and polynucleotide kinase (4GP7) [32] using DALI and UCSF Chimera [33,34], and optimized using OpenBabel [35] and GROMACS [35]. These studies gave inconclusive results: the ATP pocket has a very open conformation and it is significantly larger than ATP, allowing for wide choice in the placement of ATP-Pγ relative to the C3′-OH group of the N-acetylglucosamine moiety of UNAG (UNAG-O3’), which ranged from 3.5–8.5 Å (Figure 1A,B, Supplemental Movie S1). Thus, we decided to use molecular docking to search for better conformations.

Prior to molecular docking, the ligands were optimized using semi-empirical Quantum Mechanics (SQM) and Quantum Molecular Dynamics (QMD) with Gabedit [36] and OpenMopac [37] conducting a conformational search in vacuo and in solution. Interestingly, ATP tended to adopt a conformation between L-shaped (with the triphosphate tail rising near perpendicularly to the nucleotide ring), and a more compact, bent “scorpion-like” structure (Movie S1). Docking with Autodock Vina [38] showed a distance between ATP-Pγ and UNAG-O3’ of 3–8 Å, and the conformers produced by DOCK6 [38] showed a distance of 3.5–6.5 Å. Further refinement using molecular dynamics (MD) simulations in GROMACS [39] showed that ATP tends to stay less time in a configuration near (~3.5 Å, ~7.4% of the time) than far (>4.5 Å, 92.6% of the time) from UNAG, with several intervening water molecules, which tend to be temporarily trapped close to the Pγ of ATP. About 75.5% of the time, the distance between ATP-Pγ and UNAG-O3’ was > 5 Å apart (Movie S1). Representative conformers were selected to compare ligand–protein affinities using Xscore [40] and DrugScoreDSX [41]. Affinity predictions showed a greater affinity for ATP and UNAG-P than for ADP and UNAG (Table 1).

### 2.2. Toxin ζ Is a UNAG-Dependent ATPase

**Figure 2:**
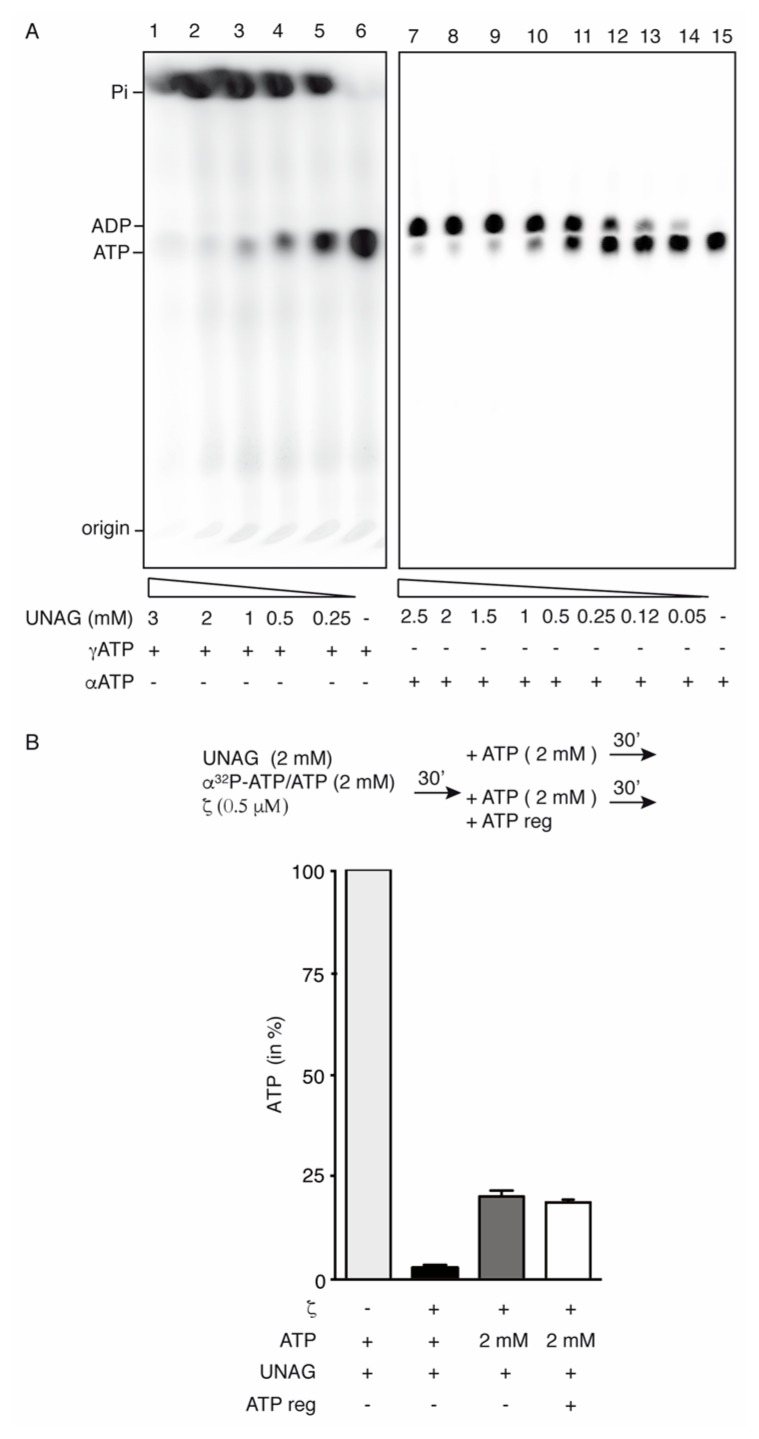
Toxin ζ hydrolyzes ATP in presence of UNAG. (**A**) Toxin ζ (500 nM), 2 mM of ATP (with fixed concentration of [ α^32^P]-ATP or [γ^32^P]-ATP, at a 1:100,000 ratio) and decreasing concentration of UNAG were incubated for 60 min at 30 °C in buffer D. ATP hydrolysis was analyzed by TLC performed on PEI cellulose plates in buffer E as the mobile phase. As control in lanes 6 and 15 UNAG was omitted. (**B**) Schematic representation of the reaction. Toxin ζ (500 nM) was incubated with UNAG (2 mM) and ATP (2 mM, with a fixed concentration of [α^32^P]-ATP, 10 nM) for 30 min in buffer D. Then, the reaction was divided in three parts. One aliquot was loaded onto a TLC. To the second and third aliquots, ATP or ATP and a regeneration system was added, and the reaction incubated for 60 min at 30 °C in buffer D. The graph represents the percentage of remaining ATP in the different conditions. The reaction without ζ was used as control. The results are expressed as the mean ± SEM of >3 independent experiments. The + and - symbols denote presence or the absence of the indicated compound.

To test whether the hydrolysis of ATP by toxin *ζ* is more efficient than the phosphotransfer of P_i_ to UNAG, the protein was incubated with its potential substrates and the products were analyzed. In the absence of UNAG, toxin *ζ* (500 nM) did not hydrolyze ATP (Figure 2A, lanes 6 and 15) [24,25]. In the presence of 1 mM UNAG, toxin *ζ* (500 nM), at half of its physiological concentration [24], hydrolyzed ~95% of the ATP substrate in a 60 min reaction (Figure 2A, lanes 3 and 10).

In the presence of limited *ζ* (60 nM), the reaction reached a steady state rate of ATP hydrolysis near the previously observed K_cat_ of ~1350 ± 165 min*^−^*^1^ (Supplementary Figure S1A,B). Previously, it has been shown that incubation of toxin *ζ* with its cognate *ε*_2_ antitoxin for 5 min at *ζ*:*ε*_2_ ratios of 1:1, inhibits *ζ*-mediated ATP hydrolysis in the presence of saturating UNAG concentrations, suggesting that the *ε*_2_ antitoxin is necessary and sufficient to inactivate toxin *ζ* [25].

A distant member of the *ζ*/PezT toxin superfamily (toxin AvrRxo1) has been proposed to transfer the ATP-Pγ to single-stranded (ss) or double-stranded (ds) DNA [42]. When UNAG was replaced by ssDNA or dsDNA, toxin *ζ* did not catalyze [γ-^32^P]-ATP/ATP hydrolysis (Figure S1B), suggesting that ssDNA or dsDNA are not the target of the *ζ* toxin. Indeed, no radiolabeled linear ssDNA or dsDNA was detected.

In the presence of increasing UNAG and fixed ATP concentrations, purified toxin *ζ* (500 nM) hydrolyzed [γ^32^P]-ATP/ATP producing ^32^P_i_/P_i_ and ADP, with ^32^P_i_/P_i_ co-migrating with the front in our experimental conditions (Figure 2A, lanes 1–5). More than 95% of the 2 mM [γ^32^P]-ATP/ATP was converted to product in 60 min (Figure 2A, lane 1). If the Pγ phosphotransfer reaction was coupled to ATP hydrolysis, a large fraction of UNAG should be converted to UNAG-P. Mass spectrometry analyses confirmed that > 95% of the ATP (molecular mass 507.18 Da, observed as peaks of 506.06 and 628.13 peaks) was hydrolyzed, as judged by the reduction of ATP and its Na-bound variant, and by the increment of the ADP peak (Figure S2A,B). Concomitantly, the UNAG peak (molecular mass 607.35 Da, observed as peaks of 606.15 and 628.13 Da) was slightly reduced and small peaks corresponding to UNAG-P (molecular mass 687.35 Da, observed as 686.12 and 708.10 Da) were detected (Figure S2B), when compared with the mock reaction (no *ζ* added) (Figure S2A). Similar results have been reported previously [24,25]. It is likely that even with an ~8-fold increase in toxin *ζ* concentration UNAG phosphorylation was limited, and >20 ATP molecules were hydrolyzed for each Pγ transferred to UNAG. Toxin *ζ* is specific for UNAG, because its ATPase activity in the presence of other UDP-activated sugars (e.g., UDP-glucose and UDP-N-acetylgalactosamine) is reduced >50-fold when compared to UNAG [23,25].

When [γ^32^P]-ATP was replaced by [*α*^32^P]-ATP, only the accumulation of the [α^32^P]-ADP/ADP product was observed (Figure 2A, lanes 7–14). To test whether the UNAG-P or the ADP formed during the reaction interfere with *ζ*-mediated ATP hydrolysis, stoichiometric amounts of UNAG and [α^32^P]-ATP/ATP (2 mM) were incubated with *ζ* (500 nM) for 30 min. Toxin *ζ* hydrolyzed > 95% of the ATP substrate to convert it into [α^32^P]-ADP/ADP and P_i_ (Figure 2B). In a second step, fresh [α^32^P]-ATP/ATP (+2 mM) in the presence or the absence of an ATP regeneration system (ATP-Reg) was added to the previous reaction, and samples were further incubated for 30 min. About 80% of the newly added [*α*^32^P]-ATP/ATP was converted to product [α^32^P]-ADP/ADP and P_i_ (Figure 2B, dark grey bar). Similar results were observed when the ATP-Reg was added (Figure 2B, empty bar). In the absence of UNAG, toxin *ζ* did not hydrolyze ATP, even in the presence of the lactate dehydrogenase/pyruvate kinase ATP-Reg system (data not shown). Unlike the distantly related AvrRxo1 toxin [42], toxin *ζ* did not transfer the ATP-P*γ* to the NAD formed during ATP-regeneration. In the presence of the ATP-Reg system, the maximal rate of *ζ*-mediated ATP hydrolysis was maintained for the first 8 h, and slightly reduced after 72 h of incubation (data not shown). It is likely that: (i) toxin *ζ* is a UNAG-dependent ATPase that phosphorylates UNAG with low efficiency (up to 7% of total UNAG); (ii) the accumulation of up to 20-fold excess of ADP does not inhibit the activity of toxin *ζ*; and (iii) traces of UNAG-P marginally reduce the activity of toxin *ζ*. This is consistent with the affinity binding predictions that revealed relatively lower affinities for ADP and UNAG than for ATP and UNAG-P (Table 1).

### 2.3. Alanine Mutagenesis of Relevant Residues

**Figure 3:**
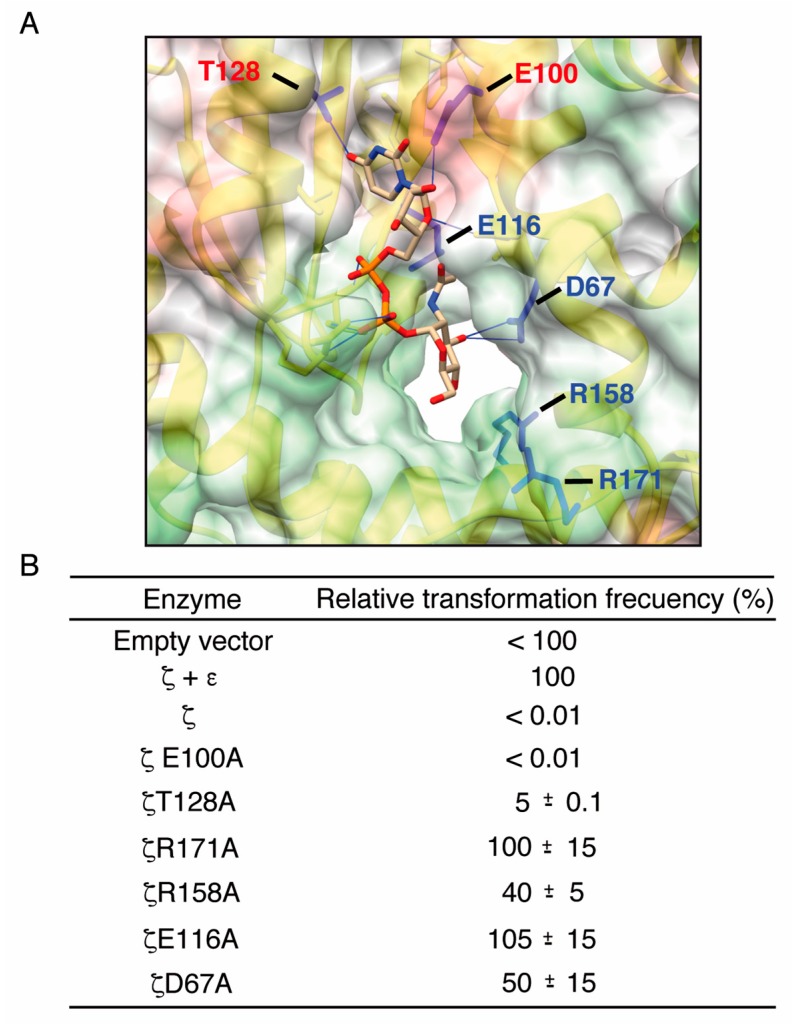
Toxin ζ residues D67, E116, R158 and R171 are relevant for enzyme activity in vivo. (**A**) Potential ζ residues relevant for UNAG binding. In blue the mutated residues that result in elimination of the toxicity of the protein; (**B**) In vivo assays for toxicity. In the absence of the antitoxin ε gene, plasmid DNA (200 ng) bearing wt ζ or its mutant variants (R171A, R158A, T128A, E116A, E100A, and D67A) were transformed into *E. coli* BL21 (DE3) [pLysS] competent cells and appropriated dilutions were platted on LB agar plates containing 500 µM IPTG. The relative transformation frequency, indicated as the mean ± SEM of > 3 independent experiments, of wt or each mutant with respect to a transformation control with plasmid-borne ζ and ε genes (100%) was represented.

From the structure of the *ζ*ε_2_*ζ*, the UNAG-*ζ*ε_2_*ζ*-UNAG complexes [19,22,23], the known structures of phosphotransferases in the ATP bound form [19,22] and the analysis of the interaction between wild type (wt) toxin *ζ* and ATP, we predicted that residues K46, D67, E100, E116, T128, R158 and R171 might play crucial roles in substrate binding. We performed an in silico alanine scanning of the *ζ*ε_2_*ζ* complex and of toxin *ζ* to understand the effect of mutations in the K46, D67, E100, E116, T128, R158 and R171 residues on UNAG binding using TRITON [43] and Modeller v9 [44]. In the toxin *ζ*-UNAG structure, residues D67, E100, E116 and T128 were in direct contact with UNAG. Residues R158 and R171, which form hydrogen bonds with the phosphate groups, might interact with Mg^2+^ and stabilize the negative charge of ATP, while K46 should interact with the β and γ phosphates of ATP (Figure 3B). The role of the *ζ*K46A mutant variant in ATP binding has been reported previously [22].

All the mutants showed a disruption of the H-bonding and contact patterns, with absence of contacts between UNAG and the mutated residue (Table 2). The effect of some of the mutants might be smaller and due to roles unrelated to UNAG binding according to affinity predictions. From the data presented in Table 2, it is likely that the role of D67 is catalytic, E116 may coordinate Mg^2+^, R158 may bind ATP, and that E100 and T128 likely have a small indirect effect through structural changes (Table 2). Residue E100 contributes to stabilize UNAG-O2’ via electrostatic and H-bonding interactions. When E100 is substituted for an alanine residue, UNAG-O2’ H-bonds with N537 instead, losing the H-bonds with R129, T118 and D67, which no longer contribute to locate UNAG in place for the transfer reaction. These changes suggest that UNAG binding and phosphorylation might be hampered in *ζ*E100A, facilitating an increase of ATP hydrolysis. The predicted binding scores of suitably oriented conformers is potentially relevant, as it may affect the efficiency of the enzyme by limiting the reaction rate through the association/dissociation rates of substrates and/or products (Table 2).

To corroborate these hypotheses experimentally, the toxin *ζ* mutant variants were constructed (Supplementary Table S1). The plasmid-bearing wt toxin *ζ*, as well as its variants lacking or containing the *ε* antitoxin gene, were purified, and then used to transform plasmid-free cells. DNA from the plasmid-borne *ε* and *ζ* genes (pCB920) transformed *E. coli* cells at a frequency arbitrarily fixed as 100% (*ζ* + *ε*_2_ condition), and with similar efficiency to the empty vector DNA (Figure 3B). In the absence of the *ε* antitoxin gene, the plasmid-borne wt *ζ* (pCB1024) and *ζ*E100A (pCB1029) did not transform *E. coli* competent cells (Figure 3B), suggesting that wt, *ζ*E100A and to a lesser extent the *ζ*T128A variant were active and toxic. When a compatible plasmid bearing the *ε* gene was present on the competent cells, the plasmid-borne wt, *ζ*E100A or *ζ*T128A genes transformed competent cells with an efficiency similar to the pCB920 control (*ζ* + *ε*_2_ condition) (data not shown).

DNA from plasmid-borne *ζ*D67A (pCB1025), *ζ*E116A (pCB1027), *ζ*R158A (pCB1028) and *ζ*R171A (pCB1030) genes efficiently transformed competent cells in the absence of the *ε* antitoxin gene (Figure 3B), suggesting that the D67, E116A, R158 and R171 residues contribute to the lethal toxin phenotype. Similar results are observed when the lysine in the predicted Walker A motif, responsible for ATP binding, was analyzed. The *ζ*K46A mutant variant lost its toxicity [22].

### 2.4. Toxin ζ Has a Low Affinity for UNAG

**Figure 4:**
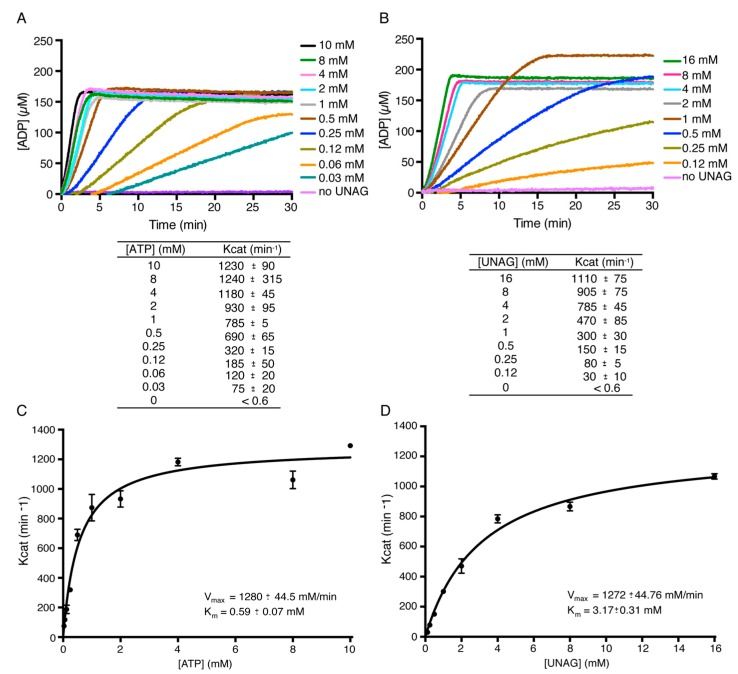
Toxin ζ-mediated ATP hydrolysis as a function of ATP and UNAG concentrations. (**A**) Toxin ζ (60 nM) was incubated for 30 min at 37 °C in buffer D containing 10 mM UNAG and the indicated amount of ATP and hydrolysis was monitored over a 30 min period. (**B**) ATP hydrolysis observed with 10 mM ATP and a variable concentration of UNAG. (**C**,**D**) Michaelis-Menten kinetic analyses of ζ for ATP and UNAG were plotted from data derived from (**A**,**B**). Data points are the mean ± SEM of >3 independent experiments. The K_cat_ for each condition is represented in the tables above the graphics.

To verify the affinity binding predictions, we examined the rate-limiting step(s) within the ATP hydrolysis cycle and performed classic Michaelis-Menten analysis to define the K_m_, K_cat_ and V_max_ (Figure 4A–D). In *B. subtilis*, the intracellular ATP and UNAG pools approached ~10 mM each in glucose-fed exponentially grown cells [13,45,46]. Similar concentrations were observed in exponentially *E. coli* K-12 cells grown in LB medium [47]. The UNAG pool, however, is significantly lower (~1 mM) in *E. coli* B cells grown in LB medium [48]. In the presence of variable ATP as the main substrate (0.03 to 10 mM) and physiological UNAG concentrations (10 mM), limiting toxin *ζ* approached the maximal rate of ATP hydrolysis K_cat_ of 1200 ± 90 min^−^^1^ (Figure 4A), with a K_m_ for ATP of ~0.6 mM (Figure 4C). This is consistent with the observation that toxin *ζ* expression, at or near physiological concentration (~1 μM), reduces the ATP pool ~2.8-fold.

In the presence of variable concentrations of UNAG (0.12 to 16 mM) and physiological ATP concentrations (10 mM), the rate of *ζ*-mediated ATP hydrolysis approached the maximal rate of ATP hydrolysis with a K_cat_ of 1100 ± 75 min*^−^*^1^ (Figure 4B), with a K_m_ for UNAG of ~3 mM (Figure 4D). Toxin *ζ* had a ~6-fold higher catalytic efficiency at 8 mM ATP (3.4 × 10^4^ M^−^^1^ s^−^^1^) than at 16 mM UNAG (5.7 × 10^3^ M^−^^1^ s^−^^1^) (Figure 4C,D), being both, ATP and UNAG concentrations, above the K_m_ value. It is likely that the significant drop in the ATP pool upon toxin *ζ* expression, its lower affinity for UNAG and the higher affinity of toxin *ζ* for UNAG-P may compromise the transfer of the P*γ* of ATP to the O3’ of UNAG and lead to a significant drop in the ATP pool. This is consistent with the affinity binding predictions that revealed relatively higher affinities for UNAG than for ATP (Table 1) and the decrease in vivo pool of the ATP pool upon toxin *ζ* expression [13]. Some of these features are also observed in a distantly related *ζ*-like toxin prevalent in Neisseria Gonorrhoeae plasmids (termed ng_*ζ*1) [49]. This new subclass of *ζ*-like toxin produces ADP in excess with respect to UNAG-P, and shows a catalytic efficiency ~8-fold higher for ATP than for UNAG [49]. Toxin ng_*ζ*1, however, phosphorylates the C4′-OH group of the N-acetylglucosamine moiety of UNAG rather than the C3′-OH group as shown for the Firmicutes toxins [18,49].

### 2.5. Toxin ζ Variants Have a Low In Vitro Activity

**Figure 5:**
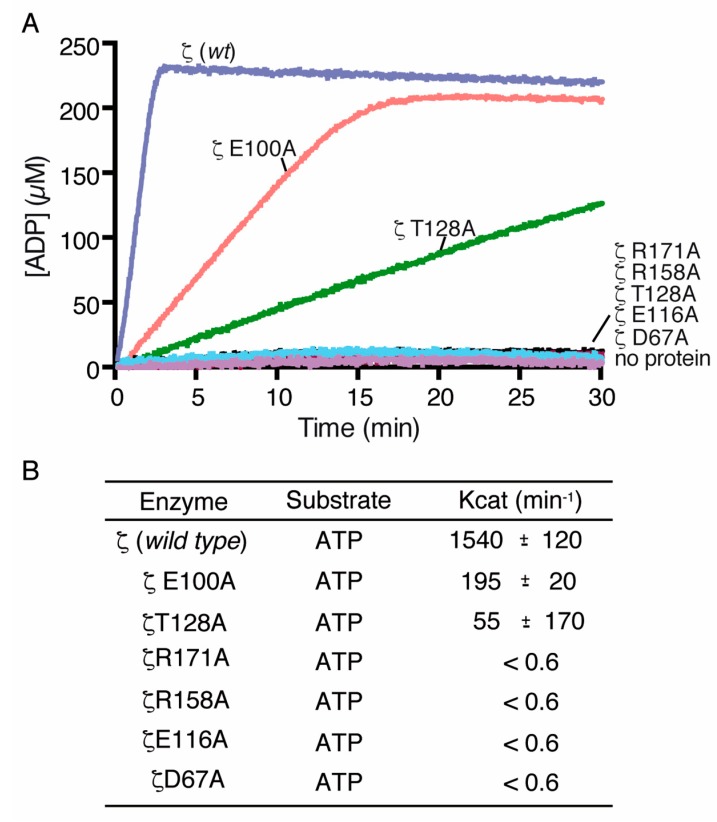
ATPase activity of toxin ζ mutants D67A, E100A, E116A, T128A, R158A and R171A. (**A**) Toxin ζ and its variants (60 nM) were incubated (30 min, 37 °C) in buffer D containing 10 mM UNAG and 10 mM ATP and ATP hydrolysis was monitored using the Shimadzu CPS-240 A dual-beam spectrophotometer; (**B**) The ATP hydrolysis rate was calculated. Data points are the mean ± SEM of >3 independent experiments.

To further explore the effect of mutations in the ATP and UNAG binding pocket, we tested the UNAG-dependent ATPase activity of the toxin *ζ* variants. The *ζ*D67A, *ζ*E100A, *ζ*E116A, *ζ*T128A, *ζ*R158A and *ζ*R171A proteins were purified using a similar protocol as that described for wt *ζ*, and biochemically characterized (Figure 5A,B). When the wt toxin *ζ* was replaced by the *ζ*E100A variant, the final steady state rate of ATP hydrolysis was reduced ~8-fold (Figure 5A,B), and this reduction in activity was still sufficient to reveal its toxic in vivo effect (see Figure 3B). When the wt toxin *ζ* was replaced by the *ζ*T128A variant, the final steady-state rate of ATP hydrolysis was reduced ~30-fold, (Figure 5A,B), but this was still sufficient to reveal a partial toxicity in vivo (see Figure 3B).

ATP hydrolysis was not observed when the wt *ζ* toxin was replaced by the *ζ*D67A, *ζ*E116A, *ζ*R158A or *ζ*R171A variants (Figure 5A,B). Similar results were observed when wt *ζ* toxin was replaced by *ζ*K46A mutant variant [22]. The presence of a mutant variant together with wt *ζ* did not affect the maximal rate of ATP hydrolysis of the latter; thus, we ruled out any inhibitory activity in the reaction mixture. Interestingly, the interactions between ATP, UNAG and the reportedly important catalytic residues are preserved in all predicted bound conformations (see Figure 1A,B) due to the flexibility of the protein.

### 2.6. Reaction Mechanism

To understand the *ζ* reaction mechanism, we modeled the reaction using the substrates alone, in solution, and in the protein moiety. Starting structures were selected from MD simulations in water and in 140 mM NaCl. These structures were further refined using OpenMopac [37]. The reaction mechanisms of *ζ* were modeled using Quantum Mechanics/Molecular Mechanics (QM/MM) with GTKDynamo [50] and pDynamo [51]. A detailed analysis of the phosphotransfer reaction using up to 140 QM steps, of 0.05 Å, starting from different initial conformations (depending on the relative positions of ATP-P*γ* and UNAG-O3’) was carried out (Table 3, Movie S1). The QM/MM models used a QM region including completely both ligands (ATP and UNAG), Mg^2+^, and intervening water (when required), instead of just the methyl-triphosphate fragment of ATP and the reacting atoms of the receiving molecule normally used [52], and the protein and nearby surrounding water using MM. Use of SQM methods allowed us to include system interactions well beyond the reach of other approaches with similar accuracy (see [53,54,55,56]).

#### 2.6.1. One Step Phosphotransference Reaction

At the physicochemical level, phosphotransferases may facilitate phosphotransfer through a variety of alternate and/or overlapping mechanisms, sometimes with more than one being feasible in a given enzyme-substrate complex (see [57,58]). To understand the structural basis for the mode of action of toxin *ζ*, we should consider various endothermic phosphorylation alternatives (see [52,59,60]).

One-step simulations model a reaction where the Pγ of ATP is directly transferred to O3’ of UNAG (using either an associative or dissociative mechanism). When the simulations started with both ligands close to each other, with no intervening water, all reactions proceeded through an associative path. In the presence of toxin *ζ* and NaCl, several Na^+^ ions cluster close to the triphosphate chain of ATP, balancing its negative charges, and the reaction proceeded with a lowering of the activation energy to ΔE^‡^ = 3.0 and of total energy release, ΔE° = −4.6 (Table 3). When the simulations started with both ligands in distant positions, the reaction mechanisms were less clear. The energy landscape varied considerably, but it was generally unfavorable (Movie S1).

#### 2.6.2. Two Step Water-Mediated Reaction

**Figure 6:**
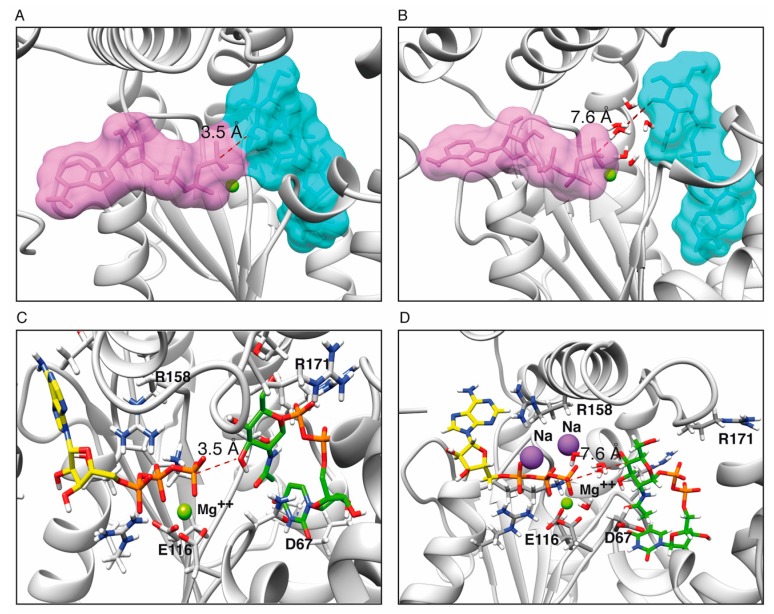
Models of toxin ζ active site with ATP·Mg^2+^ (yellow-orange) located in near and far conformations from UNAG-O3’ (green-orange). ATP-Pγ distances of 3.5 Å (**A**,**C**) and 7.6 Å (**B**,**D**) with UNAG-O3’. The water molecules and Na^+^ ions (purple ball) trapped between them are shown. The Mg^2+^ ion is depicted as a green ball.

Two-step simulations explore a possible transfer of Pγ to an intervening water followed by attack on UNAG (i.e., 1: ATP + H_2_O → ADP + Pi followed by 2: Pi + UNAG →UNAGP + H_2_O) (Table 3). The simulations only in water showed that hydrolysis of ATP, when ATP is located far from UNAG, is possible with some nearby water molecules and favorably exergonic, and that the subsequent attack on UNAG may also be favorable (Table 3). Simulations in 140 mM NaCl, whether the protein was included or not, however, showed favorable energy landscapes for ATP hydrolysis by water, but subsequent attack on UNAG by P_i_ was disfavored in all cases (Figure 6A–D, Movie S1).

## 3. Discussion

This study provides new insights that allow us to unravel the mechanisms underlying the mode of action of the toxin *ζ*. In silico modeling predicts a large ATP binding pocket. Direct modeling of how ATP could be transferred to toxin *ζ*, deduced from various related proteins, failed to predict a preferred conformation, although any of them would be precluded by formation of the *ζ*ε_2_*ζ* complex, supporting a role of ε_2_ as an inhibitor of ATP binding [22]. Further attempts to identify a preferred bound conformation for ATP using docking failed to single one out. We refined the solution structures of the ligands using QM to better understand their chemical properties. Based on extensive QM calculations, we propose here a previously unreported structure for ATP in solution. Further attempts to identify a preferred bound conformation for ATP using docking failed to single out a preferred one: all alternate locations identified for ATP maintain the same interactions with toxin *ζ*. Using bound ligand structures, we determined relative affinities of ATP, ADP, UNAG and UNAG-P for toxin *ζ*, predicting a higher turnover rate for ADP and UNAG than for ATP and UNAG-P. This allowed us to elaborate predictions for in silico mutagenesis and assign putative roles to key amino acids. Since equimolar or significant phosphate transfer from ATP-Pγ to the O3’-UNAG was not observed (Figure S2b), and toxin *ζ* is anticipated to be saturated with both ATP and UNAG, this suggests that the closest conformers may be underrepresented. This is in agreement with MD simulations, where ATP spent more time in a location far from UNAG.

Our genetic and biochemical analyses do not favor an associative mechanism for ATP hydrolysis followed by phosphorylation of a fraction of UNAG [13,14,25]. Direct quantification of the fraction of UNAG-P produced under our in vitro conditions revealed that <7% of the total UNAG substrate might be phosphorylated. This is in remarkable agreement with in silico predictions. Therefore, we favor that toxin *ζ*, as a UNAG-dependent ATPase, regulates and modulates the ATP and the UNAG pools differently. We cannot discriminate whether toxin *ζ* transfers the Pγ of ATP to a fraction of UNAG via a dissociative mechanism or by a two-step mechanism, but we can state that toxin *ζ* is mainly an UNAG-dependent ATPase and with low efficiency an UNAG phosphorylase. Our results indicate that *ζ* accomplishes its role by a combination of several mechanisms: first, it modifies the conformation of ligands in the bound state and during the reaction to a structure that is more favorable than the normal structure in solution; second the presence of water in an open active site further helps to reduce the activation energy, Na^+^ weakens the P_β_-Pγ bond attracting the dissociated Pγ; and finally the presence of the protein itself further reduces the activation energy, likely by stabilizing the transition state (Movie S1). In summary, in silico simulations favor a preferred role for toxin *ζ* as ATPase over UNAG phosphorylation. This is consistent with higher affinity for ATP and UNAG-P, and with ATP tending to spend more time staying far away from UNAG, so that ATP hydrolysis is favored, rather than close, where it would phosphorylate UNAG.

Our data are consistent with the observation that toxin *ζ* expression, at or near physiological concentration, reduces the ATP pool, leading to an imbalance of ATP/GTP ratios, which subsequently dysregulates transcription of ~78 genes [13,14]. Within 15 min of toxin expression, reversible dormancy is induced [13,14]. Within the 15–60 min interval, toxin *ζ* inhibits DNA, RNA and proteins synthesis, and later (60–90 min) toxin *ζ* transfers a fraction of the ATP-Pγ to UNAG, producing dead-end UNAG-P [13,14,24,25]. Expression of antitoxin ε_2_, even after >8 h of toxin action, reverses the *ζ* effect and cells recover their proliferation capacity [13,14,25,27], suggesting that UNAG-P might not compromise cell wall biosynthesis. This is consistent with the observation that: (i) fosfomycin, an antibiotic which inhibits MurA activity, sensitizes cells exposed to toxin *ζ* action by >20-fold [24]; and a 10-fold lower intracellular UNAG pool is reported for *E. coli* B [48] when compared with *E. coli* K12 cells [47], suggesting that dropping the UNAG pool might be compatible with normal cell proliferation. These results illustrate the catalytic roles of the toxin *ζ*, which does not deplete the bacterium of peptidoglycan precursors for de novo cell wall biosynthesis, but depletes purine nucleotides, and pave the way for future biotechnological studies addressing its activity.

## 4. Materials and Methods

### 4.1. Bacterial Strains and Plasmids

The bacterial strains and plasmids used in this study are listed in Table S1. The toxin ζ gene was over-expressed in *E. coli* BL21(DE3) cells harboring pCB920 under the control of a rifampicin-resistant promoter (P_T7_) and the ε gene under the control of a rifampicin-sensitive one (P_ω_) [13]. Mutant toxin variants D67A, E100A, E116A, T128A, R158A and R171A of toxin ζ were constructed using the mutagenic primers described in Table S2 and mutagenized as described [61]. A PCR fragment (*Pst*I/*Bam*HI) containing the specific mutation was used to replace the wt *Pst*I/*Bam*HI fragment leading to plasmids pCB925 (D67A), pCB926 (E100A), pCB927 (E116A), pCB928 (T128A), pCB929 (R158A) and pCB930 (R171A) (Tables S1 and S2).

For transformation assay the pCB920 or pCB925-to-pCB930 variants lacking the ε gene (pCB1024-to-pCB1030 DNA, 200 ng) were used to transform *E. coli* BL21 (DE3). Appropriate dilutions were plated on LB agar plates in the presence and absence of 500 μM IPTG, to measure the number of colony-forming units.

### 4.2. Biochemical Assays

*Streptococcus pyogenes* wt toxin ζ and its mutant variants were over-expressed in *E. coli* BL21(DE3) cells in the presence of rifampicin and purified. Toxin ζ, free of the ε_2_ antitoxin, was purified as reported [24]. Toxin ζ variants ζD67A, ζE100A, ζE116A, ζT128A, ζR158A and ζR171A were purified in two steps using a similar protocol [24]. In short, cells were broken using a French Press in buffer A (50 mM phosphate buffer pH 7.5, 100 mM NaCl, 5% glycerol). The soluble toxin variants were bound to a Ni-NTA column, and eluted using an imidazole gradient (2 to 75 mM). In a second step, protein containing fractions were diluted to 25 mM NaCl and passed through a Q Sepharose column. Proteins were eluted in buffer B (50 mM Tris HCl pH7.5) containing a gradient of NaCl (25 to 150 mM). The fractions containing ζ, ζD67A, ζE100A, ζE116A, ζT128A, ζR158A and ζR171A were dialyzed against buffer C (50 mM Tris HCl pH7.5, 100 mM NaCl) containing 50% glycerol and stored at −20 °C.

Thin layer chromatography (TLC) assays were used to measure the ATPase activity of the purified toxin ζ. The toxin was incubated using either fixed or increasing UNAG concentrations in buffer D (50 mM Tris-HCl pH 7.5, 50 mM NaCl, 1 mM MgCl_2_) at 30 °C. The toxin ζ concentration chosen for the TLC assays was the one that degraded 95% of the ATP substrate in 60 min at 30 °C. The reaction was started adding radiolabeled 2 mM ATP ([α^32^P]-ATP/ATP or [γ^32^P]-ATP/ATP at a 1:100,000 ratio), and stopped after 60 min by adding 25 mM EDTA. From each reaction 2–5 μL were spotted in 20 × 20 cm TLC PEI cellulose plates, and chromatography was performed for 120 min in a TLC chamber containing running buffer E [0.85 M KH_2_PO_4_ (pH 3.4)] as eluent. Dried TLC plates were analyzed with a PMI molecular imager (BioRad, Hercules, CA, USA).

The ATPase activity of wt toxin ζ and its variants (ζD67A, ζE100A, ζE116A, ζT128A, ζR158A and ζR171A) was also measured using an ATP/NADH coupled assay [62,63]. The toxin was incubated with increasing ATP concentrations in the presence of a fixed UNAG (10 mM) or fixed ATP (10 mM) and variable of UNAG for the indicated times, in buffer D (50 mM Tris-HCl pH 7.5, 50 mM NaCl, 1 mM MgCl_2_). The reactions additionally contained the NADH enzyme mix (300 μM NADH, 100 U/mL of lactate dehydrogenase, 500 U/mL pyruvate kinase, and 2.5 mM phosphoenolpyruvate) in buffer D and had a volume of 50 μL. The ATPase activity was determined monitoring the disappearance of absorbance at 340 nm, due to NADH conversion to NAD, using a Shimadzu CPS-20A dual-beam spectrophotometer. A standard curve with known amounts of NADH was obtained and used to convert the drop-in absorbance/time to ADP concentration/time [63]. V_max_ and K_m_ values were calculated by constructing Michaelis-Menten plots using R and the Graphpad Prism Software (6.0).

### 4.3. In Silico Analyses

The models of full-length of toxin ζ models bound to UNAG and ATP were built using I-TASSER with ab initio structure prediction [28]. Pockets in the protein surface were identified using 3 V [29]. Initial ligand–protein conformations were assigned by transference from related structures using Dali [33] and UCSF Chimera [34]. The complexes were initially minimized using OpenBabel [35] with the UFF, MMFF94S and GAFF force fields (4000 minimization steps), and further optimized using GROMACS with the AMBER and OPLS-AA force fields [64,65,66]. In silico alanine scanning of toxin ζ and its variants were generated with TRITON [43], and Modeller v9 [44] using a very large MD refinement.

The structures of toxin ζ substrates (ATP, ADP, UNAG and UNAG-P) were optimized with a SQM conformational search using Gabedit [36] and OpenMopac [37] with the PM7 parameter set and an implicit solvent model.

Additional protein–ligand conformations were generated using molecular docking with DOCK6 and AutoDock VINA [38], and the complexes were optimized as described. Ligand-protein affinities of selected conformers were estimated using Xscore [40] and DrugScoreDSX [41].

For MD simulations, the target temperature and pressure were 310 °K and 1 bar. Structures were subjected to an initial optimization step (up to 10,000 steps), followed by NVT ensemble (200 ps, in 10^5^ 2fs steps) using the V-rescale thermostat and an in NPT equilibrium for 200 ps (in 10^5^ 2fs steps) with the V-rescale thermostat and the isotropic Parrinello-Raman barostat. The MD simulations were also run (4 ns, in 25·10^5^ 2fs steps) using the Nosé-Hoover thermostat and the isotropic Parrinello-Raman barostat using GROMACS [39] with the AMBER and OPLS-AA force fields, and the ligand parameters were generated with ACPYPE [66] using AM1-BCC charges [64].

Detailed reaction simulations were performed using GTKDynamo [50] and pDynamo [51]. The most promising models were first refined using QM with OpenMopac following a protocol consisting of H assignment, H optimization and self-referenced optimization of the protein structure, and then subjected to a QM/MM simulation. The complete systems consisted of the ligands alone and in solution and of the protein and ligands surrounded by 10 Å of water or NaCl solution, partitioned into a QM region (complete ligands, nearby residues and relevant solvent molecules), a MM region (defined as the whole protein and all solvent molecules within 4 Å of the protein, and a fixed region (defined as solvent molecules beyond the MM limit). The reaction was modeled in the QM region approaching the reactants in 0.05 Å steps, using SQM with the PM6 parameter set and considering the appropriate charges for the system. The MM region used the AMBER force field, with parameters for the ligands being generated with GTKDynamo [50] and Ambertools [65]. Results were visualized using TRITON, UCSF Chimera, PyMol (www.pymol.org) [67], Jmol [68] and POVray (www.povray.org).

## Figures and Tables

**Table 1:** Predicted interaction between ζ and its ligands.

Condition	ATP	ADP	UNAG	UNAG-3P
H-bonds	4	1	5	8
Contacts	12	7	14	16
Atom contacts	92	35	60	82
DSX	−110.835	−61.036	−99.592	−119.225
Xscore	−6.73	4.57	−7.36	−7.81

H-bonds: total number of H-bonds between UNAG and ζ. Contacts: number of amino acids making contacts with substrate and products. Atom contacts: total number of contact atoms. DSX: DrugScore DSX score and Xscore: estimated binding energy in Kcal/mol (the lower scores indicate stronger binding affinity).

**Table 2:** Predicted interactions between UNAG and various ζ mutants.

Condition	wt	K46A	D67A	E100A	E116A	T128A	R158A	R171A
H-bonds	9	1	2	4	3	3	2	4
Contacts	13	10	11	13	9	8	8	12
Atom	39	41	50	46	39	32	46	53
Xscore	−7.36	−6.77	−6.87	−7.09	−8.81	−7.00	−7.17	−7.23
DSX	−99.592	−74.601	−85.754	−77.066	−79.611	−85.631	−83.521	−84.271

H-bonds, total number of H-bonds between UNAG and ζ. Contacts: number of amino acids making contacts with UNAG. Atom contacts, total number of contact atoms; Xscore, estimated binding energy in Kcal/mol (lower is better); DSX, DrugScore. In the two latter conditions the lower scores indicate stronger binding affinity.

**Table 3:** Alternative endothermic phosphorylation.

**ATP-Pγ Near to UNAG-O3’**	**ΔG^‡^ (Kcal/mol)**	**ΔG° (Kcal/mol)**
ATP + Mg^2+^ + UNAG (H_2_O)	13.81	33.63
ζ + ATP + Mg^2+^ + UNAG (H_2_O)	18.28	10.76
ATP + Mg^2+^ + UNAG (NaCl)	7.28	−17.84
ζ + ATP + Mg^2+^ + UNAG (NaCl)	2.98	−4.62
**ATP-Pγ Far from UNAG-O3’**	**ΔG^‡^ (Kcal/mol)**	**ΔG° (Kcal/mol)**
ATP + Mg^2+^ + UNAG (water)	5.45	−0.61
ATP + Mg^2+^ + UNAG + H_2_O (water)	23.98	16.05
ζ + ATP + Mg^2+^ + UNAG (water, tot)	79.35	14.5
ζ + ATP + Mg^2+^ + UNAG (water, react)	16.56	−48.29
ATP + Mg^2+^ + UNAG (NaCl)	161.57	95.3
ATP + Mg^2+^ + UNAG + H_2_O (NaCl)	23.16	9.77
ζ + ATP + Mg^2+^ + UNAG (NaCl)	38.64	8.57
**ATP-Pγ Far from UNAG-O3’ (two steps)**	**ΔG^‡^ (Kcal/mol)**	**ΔG° (Kcal/mol)**
ζ + ATP + Mg^2+^ + H_2_O^1059^ + UNAG (water, step 1)	9.61	0.68
ζ + ADP + Mg^2+^ + P_i_^1059^ + UNAG (water, step 2)	25.2	−9
ζ + ATP + Mg^2+^ + H_2_O^2259^ + UNAG (water, step 1)	36.14	2.29
ζ + ADP + Mg^2+^ + P_i_^2259^ + UNAG (water, step 2)	24.32	49.23
ζ + ATP + Mg^2+^ + H_2_O^2273^ + UNAG (water, step 1)	8.81	−11.05
ζ + ADP + Mg^2+^ + P_i_^2273^ + UNAG (water, step 2)	33.03	45.31
ζ + ATP + Mg^2+^ + H_2_O^3646^ + UNAG (water, step 1)	7.35	−14.44
ζ + ADP + Mg^2+^ + P_i_^3646^ + UNAG (water, step 2)	44.52	−22.06
ζ + ATP + Mg^2+^ + H_2_O^2204^ + UNAG (NaCl, step 1)	2	−16.86
ζ + ADP + Mg^2+^ + P_i_^2204^ + UNAG (NaCl, step 2)	54.53	52.21
ζ + ATP + Mg^2+^ + H_2_O^2637^ + UNAG (NaCl, step 1)	4	−9.12
ζ + ADP + Mg^2+^ + P_i_^2637^ + UNAG (NaCl, step 2)	46.12	47.54
ζ + ATP + Mg^2+^ + H_2_O^4589^ + UNAG (NaCl, step 1)	2.77	18.74
ζ + ADP + Mg^2+^ + P_i_^4589^ + UNAG (NaCl, step 2)	47.22	39.47

ΔG^‡^: activation energy (Kcal/mol) and ΔG°: reaction energy (Kcal/mol) of modeled reactions. Near: models starting from a position with Pγ near (~3.5 Å) O3’. Far: models starting from a conformation with Pγ far (>7 Å) from O3’. Water and 140 mM NaCl indicate models simulated in water and NaCl counterions. Tot: total energy change (including entropic changes). React: actual reaction energy (after ligand approximation). Step 1: model of the reaction ATP + H_2_O → ADP + P_i_. Step 2: model of the reaction P_i_ + UNAG → UNAG-P.

## Supplementary Materials

The following data are available online at https://zenodo.org/record/2537367#.XDgEVlI2W28, Figure S1: Increasing toxin ζ concentrations rise the rate of ATP hydrolysis, Figure S2. Toxin ζ phosphorylates a fraction of UNAG. Table S1: Bacterial strains and plasmids, Table S2: Oligonucleotides used for ζ mutagenesis. Video S1: dynamics of toxin ζ.
